# Metformin in Elderly Patients With Type 2 Diabetes and Muscle Strength, Muscle Mass, and Physical Function: Positive or Negative Correlation?

**DOI:** 10.1155/ije/6423817

**Published:** 2026-10-01

**Authors:** Chuanli Wei, Jieyuzhen Qiu, Mengjuan Xue, Bin Lu, Cuiping Jiang, Xiaoming Tao

**Affiliations:** ^1^ Department of Endocrinology, Huadong Hospital Affiliated to Fudan University, No. 221 Yan’an West Road Jing’an District, Shanghai, China, fudan.edu.cn

**Keywords:** grip strength, metformin, Type 2 diabetes

## Abstract

This study aimed to explore the association between long‐term use of metformin and muscle strength, muscle mass, and physical function, providing a basis for rational use of glucose‐lowering drugs in elderly patients with Type 2 diabetes. A total of 290 elderly patients were enrolled. Patients were screened using propensity score matching, resulting in 132 matched individuals, 66 in the metformin group (Group A) and 66 in the nonmetformin group (Group B). Group comparisons assessed the correlation between metformin and muscle strength, muscle mass, and physical function, with further stratification by glycated hemoglobin (HbA1c). Statistical methods included *t*‐tests, nonparametric tests, chi‐square tests, and logistic regression analyses. Results obtained are as follows: (1) The difference in the proportion of low grip strength between Groups A and B was statistically significant (48.5% vs. 19.7%, *p* < 0.001). (2) In HbA1c‐stratified analysis, when 7.8% ≤ HbA1c < 9.4%, Groups A and B showed statistically significant differences in skeletal muscle mass (17.2 ± 3.0 vs. 19.9 ± 4.5 kg, *p* = 0.049), relative skeletal muscle mass (6.5 ± 0.8 vs. 7.2 ± 1.1 kg/m^2^, *p* = 0.03), proportion of low grip strength (57.1% vs. 13.6%, *p* = 0.013), and proportion of low gait speed (71.4% vs. 40.9%, *p* = 0.039); when HbA1c ≥ 9.4%, significant difference in proportion of low grip strength (41.2% vs. 5.9%, *p* = 0.033) was observed between groups. This study found that long‐term metformin use in elderly patients with Type 2 diabetes is positively associated with a higher proportion of low grip strength. Particularly in patients with poor glycemic control, metformin constitutes an unfavorable factor for sarcopenia.

**Trial Registration:** ClinicalTrials.gov identifier: NCT07788378

## 1. Introduction

With the accelerating aging process in China, sarcopenia has drawn increasing attention. Sarcopenia is an age‐related geriatric syndrome characterized by reductions in muscle mass, muscle strength, and/or physical function [1]. Sarcopenia has a high prevalence, often insidious onset, and broad impacts on physiological functions. According to the 2019 report from the Asian Working Group for Sarcopenia (AWGS), sarcopenia prevalence among elderly Asians ranged from 5.5% to 25.7%, with males exhibiting higher rates (5.1%–21.0%) than females (4.1%–16.3%) [2]. Particularly among patients with diabetes, sarcopenia incidence was significantly elevated, with a prevalence of 14.8% reported in Chinese diabetic populations [3].

Sarcopenia exerts numerous adverse effects. Sarcopenia is strongly associated with adverse cardiovascular events and increased mortality [4]. Sarcopenia is linked to cancer and significantly elevates the risk of various postoperative complications in patients with gastrointestinal malignancies, including dysphagia, cognitive impairment, fractures, falls, hospitalization, and all‐cause mortality [5]. Sarcopenia and diabetes frequently coexist within the same individual, leading to multiple adverse outcomes. Sarcopenia results in declining functional flexibility and responsiveness in diabetic patients, causing gait alterations and impaired balance, limiting daily activities, and is closely linked to risks of falls, fractures, depression, frailty, and mortality [6–8]. Thus, it is critically important to treat sarcopenia alongside diabetes and avoid detrimental effects of hypoglycemic drugs on muscle.

Metformin lowers blood glucose by increasing muscle glucose uptake, inhibiting hepatic glucose output and gluconeogenesis, and promoting glucose excretion into the intestinal lumen; it also reduces the risk of diabetic cardiovascular disease, diabetic retinopathy, nephropathy, and neuropathy [9–12].

There remains controversy in current studies regarding metformin’s impact on sarcopenia. Some studies indicate that metformin may have potential beneficial effects on grip strength, skeletal muscle mass, and gait speed. Nordklint AK reported that after 18 months of metformin intervention in patients with Type 2 diabetes, bone mineral content and density were improved [13]. Qaisar R conducted a 16‐week metformin trial on older men, finding improvements in grip strength and gait speed with statistical significance (*p* < 0.05) [14]. Klimova AV found that a 6‐month metformin intervention improved physical function in patients [15]. Lyu Q’s murine study demonstrated that 5 months of metformin treatment conferred benefits and recovery effects on the gastrocnemius muscle in sarcopenia models [16]. Conversely, some studies indicate no impact of metformin on muscle mass, grip strength, or gait speed. Qaisar R reported no improvement in body composition or gait speed after 4 months of metformin use [17]. Laksmi PW found no significant difference in grip strength between groups after a 16‐week metformin intervention [18]. Koshizaka M reported that after 24 weeks of intervention with ipragliflozin or metformin in patients with Type 2 diabetes, metformin had no significant effect on muscle mass [19]. Previous studies have also elucidated mechanisms underlying metformin’s effects on muscle. According to Kang MJ, metformin induced myostatin expression in skeletal muscle cells via the adenosine 5′‐monophosphate‐activated protein kinase (AMPK)–FoxO3a–HDAC6 pathway, thereby impairing muscle function [20]. Other studies found that metformin reduced expression of mTORC1‐related genes in muscles of elderly individuals with impaired glucose tolerance [21], potentially leading to decreased muscle protein synthesis or increased autophagy [22].

Therefore, this study enrolled elderly patients with Type 2 diabetes and employed a retrospective cross‐sectional design with propensity score matching to elucidate the association between metformin and muscle strength, muscle mass, and physical function, providing evidence for rational clinical use of hypoglycemic drugs.

## 2. Study Subjects and Methods

### 2.1. Study Population

Elderly patients with Type 2 diabetes hospitalized at the Department of Endocrinology, Huadong Hospital affiliated with Fudan University, from August 1, 2018, to August 31, 2023, were enrolled. Inclusion criteria are as follows: (1) age ≥ 60 years; (2) diagnosed with Type 2 diabetes; (3) stable glucose‐lowering regimen for at least 3 months prior to admission; (4) completed bioelectrical impedance analysis (BIA), grip strength, and gait speed assessments; and (5) the reason for hospitalization was to regulate blood glucose. Exclusion criteria are as follows: (1) diabetic ketoacidosis or hyperosmolar coma; (2) severe hepatic, renal, or cardiopulmonary dysfunction, or severe gastrointestinal disease; (3) active malignant tumors; (4) use of glucocorticoids or antipsychotic drugs; (5) active infectious diseases; and (6) severe musculoskeletal diseases.

### 2.2. Research Methods

#### 2.2.1. Study Grouping

This retrospective cross‐sectional study grouped patients according to metformin use into metformin and nonmetformin groups. We identified the factors that differed by comparing the two groups. Propensity score matching was performed incorporating variables with intergroup differences as well as sex, age, height, weight, and glycated hemoglobin (HbA1c), using a caliper of 0.01. Patients using metformin were assigned to Group A, and those not using metformin were assigned to Group B. Subsequently, an analysis was conducted and confounding factors were adjusted for in the analysis. Then, the quartiles of HbA1c were stratified, and a total of four groups were formed. Refer to Figure 1.

**FIGURE 1:**
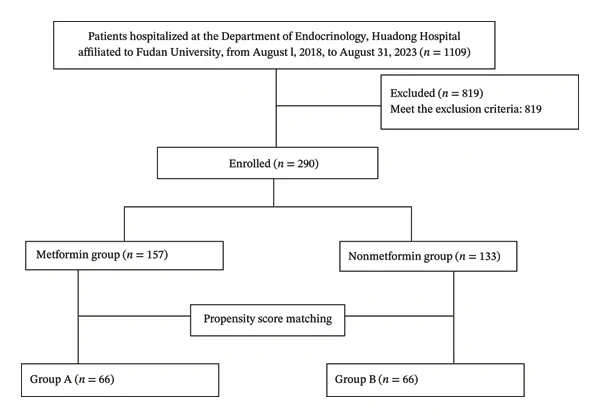
Flowchart of enrollment and grouping process.

#### 2.2.2. Criteria for Diabetes Diagnosis

According to the 1999 WHO recommended diagnostic criteria for diabetes [23]: (1) typical diabetic symptoms plus random blood glucose ≥ 11.1 mmol/L; (2) typical diabetic symptoms plus fasting blood glucose ≥ 7.0 mmol/L; and (3) typical diabetic symptoms plus 2‐h post–oral glucose tolerance test blood glucose ≥ 11.1 mmol/L. For asymptomatic individuals, confirmation by retesting on another day was required. Typical diabetic symptoms included polydipsia, polyuria, polyphagia, and unexplained weight loss; random blood glucose referred to glucose at any time of day regardless of last meal; and fasting state referred to no food intake for at least 8 h.

#### 2.2.3. Muscle Strength, Muscle Mass, and Physical Function

Grip strength was assessed using an electronic dynamometer (EH101 Model, Guangdong Senssun Weighing Apparatus Group Ltd.), measured twice for each hand with averages calculated; the maximal value from either hand was taken as the final grip strength. Low grip strength was defined as < 28 kg for men and < 18 kg for women. Gait speed was calculated by measuring time taken to walk 50 m; speed < 1 m/s was classified as low gait speed, and speed ≥ 1 m/s was classified as normal. Appendicular skeletal muscle mass (ASM) and relative skeletal muscle mass (SMI; ASM/height^2^) were measured by BIA. Low SMI was defined as < 7.0 kg/m^2^ for men and < 5.7 kg/m^2^ for women [2].

#### 2.2.4. Laboratory Tests

Upon admission, fasting measurements including hematology, hepatic and renal function tests, lipid panel, fasting glucose, fasting C‐peptide, HbA1c, and bone metabolic markers were obtained by a blood routine analyzer and a biochemical analyzer.

#### 2.2.5. Statistical Methods

Statistical analysis was conducted using SPSS version 27.0.1. Continuous variables were expressed as mean ± SD; categorical variables were expressed as percentages. Normally distributed measurement data were analyzed with *t*‐tests, and nonnormally distributed data were analyzed with nonparametric tests; categorical data were compared by the chi‐square tests. Statistical significance was defined as *α* = 0.05, with *p* < 0.05 considered significant.

#### 2.2.6. Ethic

This research has been approved by the Ethics Committee of Huadong Hospital, Fudan University, with Approval Number 2025K371.

## 3. Results

### 3.1. Baseline Characteristics

A total of 290 patients with Type 2 diabetes meeting inclusion and exclusion criteria were enrolled. Among them, 157 patients were in the metformin group and 133 in the nonmetformin group. The metformin group had been using metformin for more than 3 months, with the dosage ranging from 500 to 2000 mg. General characteristics of both groups are shown in Table 1. In Groups A and B, significant differences were found in age, height, ASM (19.8 ± 4.2 kg vs. 18.6 ± 4.5 kg), glycated albumin, aspartate aminotransferase, albumin, alkaline phosphatase, total cholesterol, blood urea nitrogen, calcium, phosphorus, high‐density lipoprotein, Type I collagen amino‐terminal peptide 2, parathyroid hormone, and N‐terminal osteocalcin (*p* < 0.05). However, no significant differences were observed in sex, body mass index (BMI), SMI, grip strength, gait speed, HbA1c, estimated glomerular filtration rate (eGFR), fasting glucose, or fasting C‐peptide (*p* > 0.05) (Table 1).

**TABLE 1:** Comparison of baseline characteristics between groups.

	Metformin group (*n* = 157)	Nonmetformin group (*n* = 133)	*p*‐Value
Sex	Male: 87 (55.4%) Female: 70 (44.6%)	Male: 62 (46.6%) Female: 71 (53.4%)	0.135

Age (years)	69.1 ± 6.7	70.8 ± 7.0	0.032

Height (cm)	165.4 ± 8.1	162.3 ± 8.4	0.002

BMI (kg/m^2^)	24.1 ± 2.8	23.6 ± 2.9	0.085

ASM (kg)	19.8 ± 4.2	18.6 ± 4.5	0.022

SMI (kg/m^2^)	7.14 ± 0.99	7.14 ± 0.99	0.133

Grip strength (kg)	25.7 ± 9.4	24.5 ± 8.7	0.266

Proportion of grip strength	Normal: 92 (58.6%) Low grip strength: 65 (41.4%)	Normal: 86 (64.7%) Low grip strength: 47 (35.3%)	0.291

Proportion of gait speed	Normal: 75 (47.8%) Low gait speed: 82 (52.2%)	Normal: 56 (42.1%) Low gait speed: 77 (57.9%)	0.334

Fasting blood glucose (mmol/L)	7.37 ± 2.84	7.20 ± 3.22	0.637

Fasting C‐peptide (ng/mL)	2.13 ± 0.99	2.03 ± 1.69	0.537

HbA1c (%)	8.17 ± 1.86	8.60 ± 1.87	0.054

Glycated albumin (%)	22.2 ± 8.4	25.1 ± 8.7	0.005

Alanine aminotransferase (U/L)	20.5 ± 16.9	21.5 ± 12.3	0.567

Aspartate aminotransferase (U/L)	18.0 ± 7.9	21.9 ± 12.8	0.002

Total protein (g/L)	66.3 ± 5.6	66.7 ± 6.4	0.54

Albumin (g/L)	41.8 ± 4.2	40.3 ± 4.00	0.002

Alkaline phosphatase (IU/L)	67.9 ± 24.3	78.4 ± 36.1	0.004

Total cholesterol (mmol/L)	4.02 ± 1.03	4.36 ± 1.28	0.013

Triglycerides (mmol/L)	1.54 ± 0.78	1.44 ± 0.87	0.31

Blood urea nitrogen (mmol/L)	5.81 ± 1.89	6.42 ± 2.38	0.017

Estimated glomerular filtration rate (mL/min)	82.6 ± 21.1	81.2 ± 29.6	0.645

Creatinine (umol/L)	79.6 ± 23.8	83.5 ± 33.7	0.257

Calcium (mmol/L)	2.28 ± 0.13	2.25 ± 0.12	0.027

Phosphorus (mmol/L)	1.16 ± 0.19	1.21 ± 0.19	0.045

Low‐density lipoprotein (mmol/L)	2.32 ± 0.86	2.54 ± 1.05	0.056

High‐density lipoprotein (mmol/L)	1.17 ± 0.27	1.32 ± 0.44	< 0.001

Type I collagen amino‐terminal peptide 2 (ng/mL)	36.5 ± 14.6	43.5 ± 18.7	< 0.001

Parathyroid hormone (pg/mL)	39.1 ± 19.3	44.5 ± 23.9	0.039

N‐Terminal osteocalcin (ng/mL)	12.7 ± 7.3	15.1 ± 6.4	0.004

*Note:* ASM: appendicular skeletal muscle mass; SMI: relative skeletal muscle mass; HbA1c: glycated hemoglobin.

Abbreviation: BMI, body mass index.

Propensity score matching: Variables with significant differences as well as sex, age, BMI, height, and HbA1c were matched, resulting in 132 successfully matched patients (66 in Group A and 66 in Group B). After matching, no baseline differences were observed except total protein (*p* = 0.038).

### 3.2. Postmatching Baseline Characteristics

After successful matching, 132 patients were included (66 in Group A and 66 in Group B). Baseline data are summarized in Table 2.

**TABLE 2:** Postmatching comparison of baseline characteristics between groups.

	Group A (*n* = 66)	Group B (*n* = 66)	*p*‐Value
Sex	Male: 32 (48.5%) Female: 34 (51.5%)	Male: 32 (48.5%) Female: 34 (51.5%)	1

Age (years)	70.0 ± 7.3	69.4 ± 6.8	0.631

Height (cm)	163.3 ± 7.8	163.5 ± 7.9	0.912

BMI (kg/m^2^)	23.8 ± 2.6	23.9 ± 2.8	0.8

ASM (kg)	18.6 ± 4.0	19.1 ± 4.3	0.486

SMI (kg/m^2^)	6.88 ± 0.94	7.04 ± 1.01	0.329

Grip strength (kg)	24.6 ± 9.6	26.5 ± 8.2	0.213

Proportion of grip strength	Normal: 34 (51.5%) Low grip strength: 32 (48.5%)	Normal: 53 (80.3%) Low grip strength: 13 (19.7%)	< 0.001

Proportion of gait speed	Normal: 27 (40.9%) Low gait speed: 39 (59.1%)	Normal: 33 (50%) Low gait speed: 33 (50%)	0.294

Fasting blood glucose (mmol/L)	7.44 ± 3.02	6.80 ± 1.96	0.152

Fasting C‐peptide (ng/mL)	2.16 ± 0.98	2.12 ± 1.30	0.849

HbA1c (%)	8.23 ± 1.98	8.21 ± 1.47	0.952

Glycated albumin (%)	22.5 ± 8.3	22.8 ± 6.1	0.799

Alanine aminotransferase (U/L)	22.2 ± 19.8	20.3 ± 10.3	0.495

Aspartate aminotransferase (U/L)	18.5 ± 8.4	19.1 ± 5.9	0.636

Total protein (g/L)	66.1 ± 5.4	68.1 ± 5.9	0.038

Albumin (g/L)	41.6 ± 3.6	41.8 ± 3.2	0.742

Alkaline phosphatase (IU/L)	71.9 ± 28.8	75.0 ± 30.2	0.546

Total cholesterol (mmol/L)	4.29 ± 1.12	4.17 ± 1.10	0.534

Triglycerides (mmol/L)	1.54 ± 0.75	1.55 ± 0.96	0.95

Blood urea nitrogen (mmol/L)	5.90 ± 1.79	6.03 ± 1.98	0.7

Estimated glomerular filtration rate (mL/min)	83.1 ± 20.3	81.2 ± 24.9	0.614

Creatinine (umol/L)	76.3 ± 18.8	83.9 ± 32.8	0.106

Calcium (mmol/L)	2.27 ± 0.12	2.29 ± 0.09	0.223

Phosphorus (mmol/L)	1.20 ± 0.18	1.20 ± 0.16	0.805

Low‐density lipoprotein (mmol/L)	2.51 ± 0.94	2.40 ± 0.89	0.487

High‐density lipoprotein (mmol/L)	1.23 ± 0.29	1.21 ± 0.29	0.693

Type I collagen amino‐terminal peptide 2 (ng/mL)	38.6 ± 13.8	40.5 ± 15.8	0.455

Parathyroid hormone (pg/mL)	40.4 ± 23.5	43.5 ± 18.1	0.405

N‐Terminal osteocalcin (ng/mL)	13.7 ± 9.0	14.3 ± 5.2	0.676

SGLT‐2 inhibitor hypoglycemic agents	9 (13.6%)	4 (6.1%)	0.144

Sulfonylurea hypoglycemic agents	19 (28.8%)	20 (30.3%)	0.849

Alpha‐glucosidase inhibitor hypoglycemic agents	15 (22.7%)	25 (37.9%)	0.058

DPP‐4 inhibitor hypoglycemic agents	17 (25.8%)	15 (22.7%)	0.685

Nonsulfonylurea insulin secretagogues	7 (10.6%)	10 (15.2%)	0.436

Insulin use	15 (22.7%)	40 (60.6%)	< 0.001

Thiazolidinedione hypoglycemic agents	3 (4.5%)	2 (3%)	1

GLP‐1 receptor agonist hypoglycemic agents	5 (7.6%)	2 (3%)	0.44

*Note:* ASM: appendicular skeletal muscle mass; SMI: relative skeletal muscle mass; HbA1c: glycated hemoglobin. SGLT‐2: sodium–glucose cotransporter 2; DPP‐4: dipeptidyl peptidase‐4; GLP‐1: glucagon‐like peptide‐1.

Abbreviation: BMI, body mass index.

Significant differences were found between groups in the proportion of low grip strength (48.5% vs. 19.7%, *p* < 0.001), total protein (*p* = 0.038), and insulin use (22.7% vs. 60.6%, *p* < 0.001). No significant differences were observed in sex, age, height, eGFR, BMI, ASM, SMI, gait speed, or HbA1c (*p* > 0.05) (Table 2).

Adjusted for age and sex, the difference in low grip strength proportion between groups was significant (48.5% vs. 19.7%, *p* < 0.001), while differences in ASM, SMI, and gait speed were not significant (*p* > 0.05) (Table 3).

**TABLE 3:** The differences in muscle phenotypes between groups after matching—unadjusted and adjusted.

	Model 1	Model 2		Model 3	
	*p*‐Value	Β‐value and 95% confidence Interval	*p*‐Value	Β‐value and 95% confidence Interval	*p*‐Value
ASM (kg)	0.486	0.926 (0.804–1.066)	0.283	0.704 (0.491–1.009)	0.056
SMI (kg/m^2^)	0.329	0.68 (0.395–1.17)	0.163	0.39 (0.144–1.058)	0.065
Grip strength (kg)	0.213	0.96 (0.911–1.012)	0.127	0.947 (0.894–1.003)	0.062
Proportion of low grip strength (%)	< 0.001	3.997 (1.791–8.922)	< 0.001	4.518 (1.814–11.249)	0.001
Proportion of low gait speed (%)	0.294	1.417 (0.695–2.887)	0.338	1.647 (0.736–3.686)	0.224

*Note:* ASM: appendicular skeletal muscle mass; SMI: relative skeletal muscle mass. Model 1—unadjusted. Model 2—adjusted for sex and age. Model 3—adjusted for sex, age, height, BMI, HbA1c, total protein, and insulin use.

After further adjustment for height, BMI, HbA1c, total protein, and insulin use, the difference in low grip strength proportion showed significance between groups (48.5% vs. 19.7%, *p* = 0.001), with no significant differences in ASM, SMI, or gait speed (*p* > 0.05) (Table 3).

When analyzing the effects of hypoglycemic drugs on muscle phenotypes, insulin use was not significantly associated with grip strength (*β* = 1.075, *p* = 0.888), gait speed (*β* = 1.24, *p* = 0.661), or SMI (*β* = 0.677, *p* = 0.519). However, metformin was significantly associated with grip strength (*β* = 4.226, *p* = 0.002) (Table 4).

**TABLE 4:** The impact of hypoglycemic drugs on grip strength, gait speed, and SMI.

	Grip strength		Gait speed		SMI	
	*p*‐Value	*β*‐Value	*p*‐Value	*β*‐Value	*p*‐Value	*β*‐Value
Metformin	0.002	4.226	0.186	1.778	0.291	1.804
SGLT‐2 inhibitor hypoglycemic agents	0.176	2.567	0.202	2.459	0.294	2.210
Sulfonylurea hypoglycemic agents	0.438	0.672	0.459	0.712	0.429	0.626
Alpha‐glucosidase inhibitor hypoglycemic agents	0.925	0.957	0.433	1.400	0.798	1.149
DPP‐4 inhibitor hypoglycemic agents	0.763	0.861	0.190	1.873	0.940	0.958
Nonsulfonylurea insulin secretagogues	0.282	1.956	0.042	4.177	0.944	0.950
Insulin use	0.888	1.075	0.661	1.240	0.519	0.677
Thiazolidinedione hypoglycemic agents	0.470	0.413	0.871	0.852	0.999	< 0.001
GLP‐1 receptor agonist hypoglycemic agents	0.128	0.171	0.231	0.330	0.999	< 0.001
Total protein	0.313	0.963	0.657	0.985	0.122	1.066

*Note:* SMI: relative skeletal muscle mass; SGLT‐2: sodium–glucose cotransporter 2; DPP‐4: dipeptidyl peptidase‐4.

Abbreviation: GLP‐1, glucagon‐like peptide‐1.

### 3.3. Stratified Analysis

A stratified study was conducted based on gender, dividing into males and females. We found that in males, there was a significant difference in proportion of low grip strength between the two groups (43.7% vs. 18.7%, *p* = 0.031) (Table 5). In females, there were significant differences in proportion of low grip strength (52.9% vs. 20.6%, *p* = 0.006) and proportion of low gait speed (64.7% vs. 38.2%, *p* = 0.029) between the two groups (Table 5).

**TABLE 5:** Postmatching comparison in males and females.

	Males			Females		
	Group A (*n* = 32)	Group B (*n* = 32)	*p*‐Value	Group A (*n* = 34)	Group B (*n* = 34)	*p*‐Value
BMI (kg/m^2^)	24.2 ± 2.3	24.1 ± 2.9	0.913	23.5 ± 2.9	23.8 ± 2.7	0.659

ASM (kg)	21.6 ± 2.8	22.6 ± 2.8	0.163	15.7 ± 2.4	15.7 ± 2.0	0.943

SMI (kg/m^2^)	7.58 ± 0.66	7.82 ± 0.69	0.152	6.22 ± 0.63	6.31 ± 0.64	0.555

Grip strength (kg)	30.6 ± 8.7	32.1 ± 6.6	0.456	18.9 ± 6.3	21.3 ± 5.9	0.11

Proportion of grip strength	Normal: 18 (56.3%) Low grip strength: 14 (43.7%)	Normal: 26 (81.3%) Low grip strength: 6 (18.7%)	0.031	Normal: 16 (47.1%) Low grip strength: 18 (52.9%)	Normal: 27 (79.4%) Low grip strength: 7 (20.6%)	0.006

Proportion of gait speed	Normal: 15 (46.9%) Low gait speed: 17 (53.1%)	Normal: 12 (37.5%) Low gait speed: 20 (62.5%)	0.448	Normal: 12 (35.3%) Low gait speed: 22 (64.7%)	Normal: 21 (61.8%) Low gait speed: 13 (38.2%)	0.029

*Note:* ASM, appendicular skeletal muscle mass; SMI, relative skeletal muscle mass.

Abbreviation: BMI, body mass index.

HbA1c was stratified by quartile into four groups: HbA1c < 7%, 7% ≤ HbA1c < 7.8%, 7.8% ≤ HbA1c < 9.4%, and HbA1c ≥ 9.4%.

In the HbA1c < 7% subgroup, Group A had 17 patients and Group B had 11 patients; in the 7% ≤ HbA1c < 7.8% subgroup, Group A had 18 patients and Group B had 16 patients. Within HbA1c < 7.8%, no differences in hypoglycemic agents between groups A and B were found (*p* > 0.05). After adjusting for age and sex, no significant differences were observed between groups in ASM, SMI, grip strength, or gait speed (*p* > 0.05). Refer to Figure 2.

**FIGURE 2:**
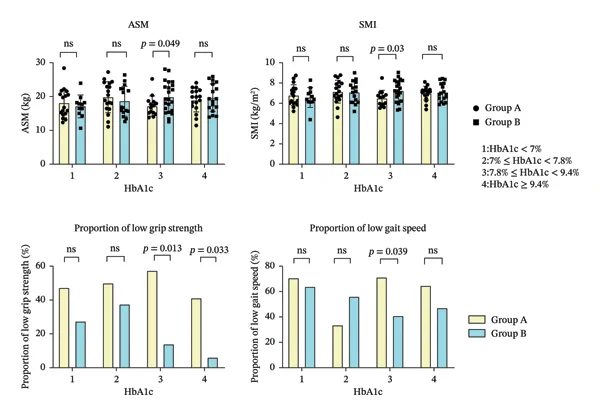
Data adjusted for age and sex; ns indicates *p* > 0.05; ASM: appendicular skeletal muscle mass; SMI: relative skeletal muscle mass; HbA1c: glycated hemoglobin.

In the 7.8% ≤ HbA1c < 9.4% subgroup, Group A had 14 patients and Group B had 22 patients. Use of various hypoglycemic drugs did not differ significantly between Groups A and B (*p* > 0.05). Significant differences between Groups A and B were found in ASM (17.2 ± 3.0 vs. 19.9 ± 4.5 kg, *p* = 0.04), SMI (6.5 ± 0.8 vs. 7.2 ± 1.1 kg/m^2^, *p* = 0.032), and proportion of low grip strength (57.1% vs. 13.6%, *p* = 0.006); gait speed differences were not significant (*p* > 0.05). Adjusted for age and sex, significant differences between groups were found in ASM (*p* = 0.049), SMI (*p* = 0.03), proportion of low grip strength (*p* = 0.013), and proportion of low gait speed (71.4% vs. 40.9%, *p* = 0.039). Refer to Figure 2.

In the HbA1c ≥ 9.4% subgroup, both groups had 17 patients each. A significant difference in insulin use was observed between groups (0% vs. 62.5%, *p* < 0.001). Proportion of low grip strength differed significantly (41.2% vs. 5.9%, *p* = 0.015) between groups, whereas no significant differences were found in ASM, SMI, or gait speed (*p* > 0.05). Adjusted for age and sex, the difference in proportion of low grip strength was significant (*p* = 0.033), with no differences in ASM, SMI, or gait speed (*p* > 0.05). Refer to Figure 2.

## 4. Discussion

This study included patients with Type 2 diabetes aged 60 years or older and analyzed the relationship between metformin and muscle strength, muscle mass, and physical function via a retrospective cross‐sectional design with propensity score matching. Groups A and B matched by propensity score showed a significant difference in proportion of low grip strength (48.5% vs. 19.7%, *p* < 0.001), whereas differences in ASM, SMI, and gait speed were nonsignificant (*p* > 0.05). Stratified by gender, we found that in males, there was a significant difference in proportion of low grip strength between the two groups (43.7% vs. 18.7%, *p* = 0.031). In females, there were significant differences in proportion of low grip strength (52.9% vs. 20.6%, *p* = 0.006) and proportion of low gait speed (64.7% vs. 38.2%, *p* = 0.029) between the two groups. Stratified by HbA1c and adjusted for sex and age, within 7.8% ≤ HbA1c < 9.4%, significant differences between groups were observed in ASM (17.2 ± 3.0 vs. 19.9 ± 4.5 kg, *p* = 0.049), SMI (6.5 ± 0.8 vs. 7.2 ± 1.1 kg/m^2^, *p* = 0.03), proportion of low grip strength (57.1% vs. 13.6%, *p* = 0.013), and proportion of low gait speed (71.4% vs. 40.9%, *p* = 0.039). For HbA1c ≥ 9.4%, the difference in proportion of low grip strength (41.2% vs. 5.9%, *p* = 0.033) was significant.

According to previous research, Type I collagen amino‐terminal peptide 2 was associated with skeletal muscle mass [24], and N‐terminal osteocalcin could serve as a marker for bone formation [25]. In our study, there was a significant difference between the metformin group and the nonmetformin group in Type I collagen amino‐terminal peptide 2 and N‐terminal osteocalcin. Therefore, by incorporating these indicators into the matching process, the impact they have on muscle function can be reduced.

We found that after propensity score matching, both unadjusted and adjusted models showed significant differences in proportion of low grip strength between groups (48.5% vs. 19.7%, *p* < 0.05), whereas no differences were found in ASM, SMI, or gait speed (*p* > 0.05), suggesting a negative association between metformin and grip strength. Both males and females showed significant differences in proportion of low grip strength between groups (*p* < 0.05). This aligned with Sexton P’s findings that 24‐week metformin treatment in patients with Type 2 diabetes and chronic obstructive pulmonary disease significantly reduced grip strength [26]. Previous studies have also elucidated mechanisms underlying metformin’s effects on muscle. According to Kang MJ, metformin induced myostatin expression in skeletal muscle cells via the AMPK–FoxO3a–HDAC6 pathway, thereby impairing muscle function [20]. Other studies found that metformin reduced expression of mTORC1‐related genes in muscles of elderly individuals with impaired glucose tolerance [21], potentially leading to decreased muscle protein synthesis or increased autophagy [22]. Previous studies have shown that metformin is rapidly transported to skeletal muscle through organic cation transporters, and it could stimulate the activity of AMPKα1 and *α*2 in both fast‐twitch and slow‐twitch muscle fiber types [27], but there were differences in insulin use between the two groups after matching, and Group B had a higher number of people using insulin. According to Sugimoto K, the use of insulin was beneficial to the muscles [28]. This was inconsistent with our research. However, it revealed the SMI of those using insulin was higher, but insulin use was not related to grip strength or gait speed. In our study, there were differences in the proportion of low grip strength between the two groups. There were no significant differences in ASM, SMI, or gait speed between the two groups. Meanwhile, after adjusting the insulin use, the results showed the same. When analyzing the effects of hypoglycemic drugs on muscle phenotypes, insulin use was not significantly associated with grip strength (*β* = 1.075, *p* = 0.888), gait speed (*β* = 1.24, *p* = 0.661), or SMI (*β* = 0.677, *p* = 0.519). Metformin was significantly associated with grip strength (*β* = 4.226, *p* = 0.002) (Table 4). However, some studies have shown that metformin is beneficial to the muscles. According to Petrocelli JJ, metformin could prevent the reduction in muscle fibers in the elderly and improve the remodeling of muscle fibers [29]. This was contrary to our research. We hypothesize that the following reasons may explain this. During the recovery process of disuse‐induced muscle atrophy, metformin may inhibit excessive inflammatory responses by activating AMPK. A basic study showed that AMPK was a potent negative regulator of core inflammatory pathways such as NF‐κB [30]. In a review article, it was mentioned that inflammation (such as NF‐κB) was an important mechanism in muscle atrophy [31]. In this case, the use of metformin can achieve the effect of promoting muscle function recovery by inhibiting inflammation. However, our research subjects are not patients with muscle atrophy. The mechanism of action of metformin may be that it can activate AMPK to inhibit mTORC1 or inhibit mTORC1 in a Rag GTPase‐dependent manner [32], thereby exerting a negative impact on the muscles.

HbA1c stratification analysis indicates that glycemic control may modulate the association between metformin and sarcopenia diagnostic components. After propensity score matching, no significant differences were observed between groups in ASM, SMI, grip strength, or gait speed when HbA1c < 7.8% (*p* > 0.05). Within the 7.8% ≤ HbA1c < 9.4% group, ASM (17.2 ± 3.0 vs. 19.9 ± 4.5 kg, *p* = 0.04), SMI (6.5 ± 0.8 vs. 7.2 ± 1.1 kg/m^2^, *p* = 0.032), and proportion of low grip strength (57.1% vs. 13.6%, *p* = 0.006) differed significantly between groups. After adjusting for age and sex, differences were significant in ASM (*p* = 0.049), SMI (*p* = 0.03), proportion of low grip strength (*p* = 0.013), and proportion of low gait speed (71.4% vs. 40.9%, *p* = 0.039). For HbA1c ≥ 9.4%, proportion of low grip strength differed significantly (41.2% vs. 5.9%, *p* = 0.015). Stratified analysis suggests that when HbA1c ≥ 7.8%, metformin negatively correlates with ASM, SMI, grip strength, and gait speed, indicating a negative association between metformin and muscle function under poor glycemic control. Metformin acts by activating AMPK, promoting translocation of glucose transporters to hepatocyte membranes, enhancing insulin receptor activity in hepatocytes, inhibiting triglyceride breakdown in adipocytes, suppressing fat accumulation in liver and skeletal cells, reducing free fatty acids in blood, alleviating inflammation induced by high‐fat diets, inhibiting intestinal glucose absorption, increasing glucose utilization in insulin‐independent tissues, and mitigating insulin resistance effects on target organs [33, 34]. In muscle, metformin increases insulin‐stimulated glucose uptake, activates the muscle AMPK pathway, inhibits fat accumulation in skeletal cells, suppresses osteoclast differentiation, and promotes osteoblast formation [35–38]. Activation of AMPK in skeletal muscle promotes catabolism and suppresses anabolic metabolism [39]. Therefore, independent of blood glucose, metformin negatively correlates with muscle function. These findings align with Sugimoto K, whose 1‐year longitudinal study in Japanese diabetic patients identified HbA1c reduction ≥ 1% as an independent predictor of skeletal muscle mass index and gait speed [28]. We speculate that patients with high HbA1c may have poor blood sugar control, which can damage muscle function. Coupled with the adverse effects of metformin on muscles, the negative impact of metformin on muscles is more pronounced in the 7.8% ≤ HbA1c < 9.4% subgroup. However, further research is still needed.

Study limitations are as follows: (1) As a retrospective cross‐sectional study, causal relationships between metformin and sarcopenia could not be established. Although propensity score matching was used to minimize confounders, unknown confounders might still exist. Thus, large‐sample prospective studies are needed to verify metformin’s impact on sarcopenia. (2) The duration of metformin use varied. It could range from 3 months to several years, and this might have an impact on the results. (3) After successful matching, there were differences in insulin use between the two groups, which might have an impact on the results. (4) Other hypoglycemic drugs might affect the results. According to a review summarizing the effects of antidiabetic drugs on skeletal muscle, insulin, thiazolidinedione drugs, glucagon‐like peptide‐1 (GLP‐1) analogs, and dipeptidyl peptidase‐4 (DPP‐4) inhibitors might be useful or have no impact on the maintenance of skeletal muscle mass, whereas the effects of sodium glucose cotransporter‐2 (SGLT2) inhibitors were unknown, and sulfonylureas and glinides might be unfavorable [40].

## 5. Conclusions

We found that prolonged metformin therapy correlated positively with a higher proportion of low grip strength among elderly patients with Type 2 diabetes. Particularly in patients with poor glycemic control, metformin was an adverse factor for sarcopenia. Thus, through this retrospective cross‐sectional study, it is suggested that muscle strength, muscle mass, and physical function, including ASM, SMI, grip strength, and gait speed, should be closely monitored in Type 2 diabetes patients using metformin, especially those with inadequate glycemic control.

## Funding

This article was supported by the Noncommunicable Chronic Diseases‐National Science and Technology Major Project (2026ZD0556900; 2026ZD0556901), the Shanghai Municipal Health Commission (2025BJPT003), and the Bethune Charitable.

## Ethics Statement

This research has been approved by the Ethics Committee of Huadong Hospital, Fudan University, with Approval Number 2025K371.

## Consent

Informed consent was obtained from all participants.

## Conflicts of Interest

The authors declare no conflicts of interest.

## Data Availability

The data that support the findings of this study are available from the corresponding author upon reasonable request.
